# Acute Hepatitis and Pancytopenia in a Child With Chronic Abuse of Senna

**DOI:** 10.7759/cureus.12436

**Published:** 2021-01-02

**Authors:** Amal Haoudar, Nabila Chekhlabi, Chafik El Kettani, Nezha Dini

**Affiliations:** 1 Critical Care Medicine, Cheikh Khalifa International University Hospital, Mohammed VI University of Health Sciences, Casablanca, MAR; 2 Paediatrics, Cheikh Khalifa International University Hospital, Mohammed VI University of Health Science, Casablanca, MAR; 3 Paediatrics, Cheikh Khalifa International University Hospital, Mohammed VI University of Health Sciences, Casablanca, MAR

**Keywords:** cassia angustifolia, acute hepatitis, pancytopenia, deep vein thrombosis

## Abstract

Long-term use of Senna and its anthraquinone glycosides has been associated with the development of hepatotoxicity in both children and adults.

Our case study aims to present, for the first time, acute hepatitis associated with pancytopenia in relation probably to liver and bone marrow toxicity in a three-year-old child suffering from chronic abuse of Senna.

We report the case of a three-year-old girl with a history of chronic constipation regularly treated with drinkable preparations made from the Senna plant and hospitalized eight months ago with an almost similar and reversible clinical presentation, probably of toxic origin. She was admitted to a pediatric intensive care unit with severe acute hepatitis and profound pancytopenia. Her first physical examination revealed an unconscious child with a Glasgow score of 11/15, generalized hypotonia, bleeding from the gum lining, facial erythrosis with peeling cheeks, hair loss (telogen effluvium), erythematous lesions ulcers of the anal margin, and fever. The myelogram performed two days after admission revealed a rich regenerative bone marrow with signs of inflammation. Besides, she developed deep vein thrombosis three days after placing her femoral catheter.

Pancytopenia in hospitalized children is a rare but alarming situation. In our case, malignancy was excluded, as well as a severe infection. The cause of pancytopenia could be related to the toxic effects of Senna. Chronic use of Senna may be associated with bone marrow and liver toxicity and lead to deep vein thrombosis.

## Introduction

Senna or *Cassia angustifolia* is a medicinal plant whose active ingredients are anthraquinone derivatives and their glucosides and is widely used for its laxative properties in cases of constipation in children [1]. The main mechanism of action of Senna is a selective action on the nerve plexus of intestinal smooth muscle which increases intestinal motility. Long-term use of Senna can lead to laxative dependence, electrolyte disturbances, and liver damage. Many clinicians avoid Senna for reasons such as tolerance or side effects, but this has little scientific justification, especially in children. Pancytopenia in children is a relatively rare phenomenon encountered in clinical practice. Identifying the underlying cause of pancytopenia requires a comprehensive approach and can be difficult given the wide range of etiologies, including iatrogenic causes (herbal remedies, drugs), autoimmune diseases, malignant tumors, infections, hemophagocytosis, and inherited diseases [2].

The aim of our case report is to present for the first time acute hepatitis associated with deep pancytopenia and venous thrombosis in a three-year-old child suffering from chronic abuse of Senna.

## Case presentation

A three-year-old girl was referred from Niger to the Cheikh Khalifa International University Hospital for hepatic encephalopathy and deep pancytopenia progressing for three days before her admission. She had a pathological history of chronic constipation since the age of 18 months, treated regularly with large amounts of oral Senna preparations (2-3 g of dry Senna leaves added to a cup of water, given to the patient more than three times a week). She had previously been hospitalized eight months ago, in another hospital structure, for a severe clinical picture of acute hepatitis and bicytopenia (thrombocytopenia and leukopenia), with a good clinical and biological outcome under intravenous corticosteroid therapy with an oral relay. A follow-up blood test taken two months after this episode was strictly normal. She had no particular family history. The story of her current illness began with the onset of sharp abdominal pain, vomiting, greenish diarrhea, anorexia, fever of 40° C, and deterioration in general condition. She was first hospitalized in Niger, where a workup was carried out showing hepatic cytolysis with negative hepatitic serologies and a blood smear negative for the malaria parasite. She received treatment based on third-generation cephalosporin and aminoglycoside. Due to the worsening of her condition, with the appearance of disturbances in consciousness, hair loss, facial erythroderma and bicytopenia in biology, she was transferred to us for continued treatment.

On admission to the pediatric intensive care unit (ICU), the child was unconscious with a Glasgow score of 11/15 and generalized hypotonia, but without sensorimotor deficit or signs of brain engagement. She was febrile at 40° C and polypneic at 40 cpm, with rhonchi in the pulmonary fields and correct saturation at 98% in ambient air. She was hemodynamically stable with a heart rate of 97 bpm and blood pressure of 118/88 mmHg. She presented with gum bleeding, mild facial erythrosis with scaly cheeks, mild jaundice, telogen effluvium, and vesicular and erythematous lesions of the anal margin. Otherwise, she had no lymphadenopathy or hepatosplenomegaly, and her throat was clean.

Her blood count revealed anemia at 9.5 g/dl, severe thrombocytopenia at 20,000/mm³, severe leukopenia at 730/mm³, with neutrophils at only 370/mm³ and lymphocytes at 300/mm³. The level of C-reactive protein had returned very high to 218 mg/L, procalcitonin had increased to 2.20 ng/mL, aspartate transaminase (AST) to 290 IU/L and alanine transaminase (**​​​​**​​​ALT) to 186 IU/L, total bilirubin level was at 34 mg/L, unconjugated bilirubin level at 9 mg/L, conjugated bilirubin level at 25 mg/L, Gamma-glutamyl transpeptidase level at 65 UI/L, alkaline phosphatase at 730 UI/L, prothrombin time at 47%, and creatinine serum level at 5.4 mg/L.

**Figure 1:**
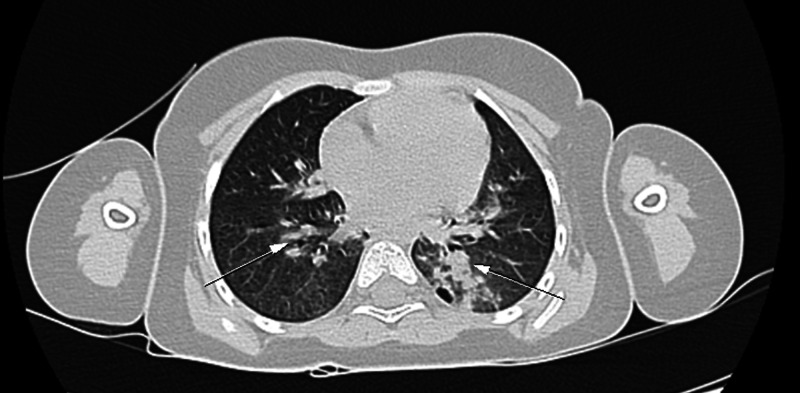
Chest CT scan showing bilateral parenchymal condensation (arrows)

**Figure 2:**
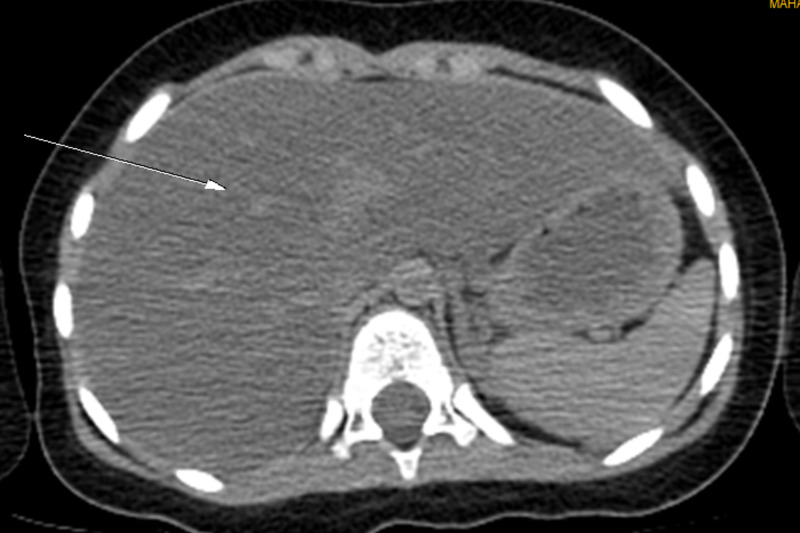
Abdominal CT scan section showing mild hepatic steatosis (arrow)

Radiologically, a brain MRI, carried out on admission, showed no abnormalities. A chest scan revealed a double focus of bilateral pneumonia (Figure 1). Abdomino-pelvic ultrasound and abdominal CT scan revealed diffuse fatty liver disease (Figure 2). The cardiac ultrasound was normal.

Regarding the infectious assessment, the urine analysis revealed hematuria, without leukocyturia or bacteria, the cytobacteriological examination of the sputum was also sterile, the thick smear test was negative, the serological tests for viral hepatitis A, B, and C were negative. The HIV serological test, screening for Epstein-Barr virus, cytomegalovirus and parvovirus B19 in blood by polymerase chain reaction (PCR) were also negative. Blood cultures on standard media and on Sabouraud agar did not identify any bacteria. Tests for autoimmune hepatitis were negative. Toxicological screening in blood and urine were negative. The inflammation assessment was not in favor of a macrophage activation syndrome, with serum fibrinogen level at 4.68 g/L, serum ferritin at 423 ng/mL, sedimentation rate at 150 mm, serum triglycerides at 1.24 g/L, and total serum cholesterol at 0.63 g/L. The myelogram, carried out on the second day of hospitalization, showed regenerative marrow with moderate inflammation. There were no signs in favor of malignancy. A liver biopsy was not done due to hemostasis disorders.

The diagnosis was acute hepatitis associated with pancytopenia, most likely toxic, based on the child's history. Admission treatment included oxygen therapy, central line placement, intravenous rehydration, broad-spectrum antibiotic therapy with ceftazidime and amikacin, because she was neutropenic and was on ceftriaxone three days earlier with persistent fever and aggravation of C-reactive protein and procalcitonin serum levels. In addition, she was given antifungal and antiviral drugs, red blood cell transfusions and platelets. The clinical course was marked by the persistence of fever and coma for the first three days with recurrent digestive bleeding and loss of all hair. On the fourth day, the child began to wake up with a regression of the fever but with the appearance of painful edema of the left lower limb. A venous Doppler ultrasound revealed the appearance of left femoro-popliteal thrombophlebitis extending to the common iliac vein. The catheter was removed, and treatment with curative-dose enoxaparin was started. Follow-up blood work showed an increase in platelets at 157,000/mm³, leukocytes at 2,227/mm³, and neutrophils at 900/mm³. C-reactive protein dropped to 30 mg/l (Table 1). The liver function test was appropriate. The clinical course was favorable, with appetite recovery and hair regrowth.

**Table 1:** Laboratory findings in the patient with pancytopenia MCV: mean cell volume; MCH: mean cell hemoglobin; MCHC: mean corpuscular hemoglobin concentration

TEST	Normal values	J1	J2	J4	J6	J7	J8	J15
Blood leukocyte count, 10ᶟ/mmᶟ	7 – 12	2.01	0.73	0.77	2.27	2.7	3.94	5.87
Lymphocyte, %	-	14.9	41.1	62.3	55.5	57	36.0	53.0
Lymphocyte count, 10ᶟ/mmᶟ	3.0 – 9.3	0.30	0.30	0.48	1.26	1.54	1.42	3.11
Neutrophil, %		82.6	50.7	10.4	39.7	33	55.8	32.3
Neutrophil count, 10ᶟ/mmᶟ	6 – 23.5	1.66	0.37	0.08	0.90	0.89	2.20	1.90
Monocyte, %	-	1.5	8.2	27.3	4.8	7.4	7.6	11.2
Monocyte count, 10ᶟ/mmᶟ	<3.5	0.03	0.06	0.21	0.11	0.2	0.30	0.66
Basophil, %	-	0.5	0.0	0.0	0.0	1.9	0.3	0.9
Basophil count, 10ᶟ/mmᶟ	<0.01	0.01	0.00	0.00	0.00	0.05	0.01	0.05
Erythrocyte count, 10¹²/L	4.5 - 7	3.93	3.57	4.00	5.55	3.69	4.12	3.49
Hemoglobin, g/dL	14 - 20	10.5	9.5	10.2	14.1	9.8	10.4	12.8
Hematocrit, %	50-60	30.4	32.6	30.7	42.8	30	31.9	37
Mean cell volume (MCV), fL	72-85	77.4	91.3	76.8	77.1	81.3	77.4	82.4
Mean cell hemoglobin (MCH), pg	27 - 32	26.7	26.6	25.5	25.4	26.6	25.2	28.5
Mean corpuscular hemoglobin concentration (MCHC), g/dL	32.0 - 36	34.5	29.1	33.2	32.9	32.7	32.6	34.6
Platelet count, 10ᶟ/mm	150 - 550	38	20	99	174	250	384	444
C-reactive protein level, mg/L	< 8	218.42	193.67	69.63	31.80	24.52	13.5	1.38
Procalcitonine, ng/ml	< 0.5	2.20	2.00	1.90	0.74	0.43	0.28	0.04
Creatinine, mg/L	6 - 12	5.40	5.80	5.94	4.97	4.1	4.48	4.5
Blood urea nitrogen, g/L	0.15 - 0.45	0.30	0.26	0.25	0.23	0.07	0.07	0.06
Alanine aminotransferase, U/L	<55	186	130	56	49	40	21	14
Aspartate amino transferase, U/L	5-34	290	220	108	93	84	61	25
Lactico deshdrogenase, UI/L	80-230	802	745	675	340	180	163	130
Fibrinogen, g/L	2-4.5	4.68	3.50	3.23	3.72	3.82	4.32	4.12
Prothrombin time, %	70-100	47	58	81	79	81	83	89
Activated partial thromboplastin time, seconds	32	34.1	30.5	24.2	25.0	22.9	33.2	35.5
Serum ferritin, ng/mL	15-80	423.61	445.62	376.87	322.45	265.81	252.53	11.97v

She was released from the hospital after 15 days and put on anticoagulant treatment for three months. A follow-up blood tests one month and three months after discharge were completely normal. Doppler control ultrasound of the femoral vein revealed complete resolution of the thrombosis three months later.

## Discussion

Senna, also called *Senna alexandrina* (or *Cassia angustifolia*) is a shrub whose twigs and flowers are used in herbal medicine to cure constipation. A powerful laxative, Senna contains active ingredients, such as natural anthraquinone derivatives, which promote the action of the intestinal flora during transit [1]. Their chronic abuse can be associated with serious manifestations, including loss of fluid and electrolytes, as well as chronic diarrhea. Severe hepatotoxicity is unusual but could be explained by the exposure of the liver to unusual amounts of toxic metabolites of anthraquinone glycosides [2]. Several clinical studies have shown that Senna is effective against occasional constipation, under medical supervision. The European Medicines Agency recommends this medicinal plant for adults and children over 12 years of age.

Senna poisoning is rarely reported in the literature and its potential for toxicity is largely underestimated. This makes it difficult to attribute often severe hepatic failure to this seemingly harmless agent. Experimental studies in animals have shown that subacute exposure to senna (10% senna in the diet) produced signs of hepatic and renal toxicity in rats [3]. In humans, few case studies of hepatitis and allergic reactions associated with excessive use of senna have been reported [2-4]. With long-term use and at higher doses of Senna, adverse events have been described, including several cases of clinically apparent liver damage. The time to onset of liver damage was generally three to five months of use. The hepatic failure was usually mild to moderate in severity and resolved quickly with the discontinuation of the treatment. In at least one case, re-exposure led to a rapid recurrence of liver damage, as was the case with our small patient [5].

In addition, a Senna-related plant commonly referred to as Coffee Senna or *Cassia occidentalis *has been associated with numerous cases of acute and severe toxicity with encephalopathy, myopathy, and hepatic dysfunction. Epidemics of the so-called "hepato-myoencephalopathy" syndrome in children in India were probably caused by the same plant, *Cassia occidentalis *[6,7].

A similar clinical presentation occurs in animals that consume a lot of *Cassia occidentalis*. It is not known whether this hepato-myoencephalopathy syndrome has a pathogenesis similar to the toxic hepatopathy attributed to typical Senna (*Cassia angustifolia*), which is used as a laxative [6].

Our patient had twice developed acute hepatitis and pancytopenia, which resolved spontaneously with no obvious etiology. After ruling out malignant blood disorders and any infectious cause, the toxic etiology linked to the Senna plant remains very probable, given its chronic consumption and all the more so as she presented during her hospitalization a symptomatic deep venous thrombosis on the central femoral catheter despite thrombocytopenia. An exceptional case of venous thrombosis has been reported in a 42-year-old woman, who developed sudden abdominal pain and was found to have portal vein thrombosis two years after the start of daily consumption of tea made from Senna leaves [8].

## Conclusions

Senna fruit is one of the most important herbal medicine used worldwide for the treatment of constipation. Chronic ingestion of Senna in children, for its laxative properties, can lead to reversible acute liver failure with encephalopathy and coagulopathy. This case suggests that excessive intake of large quantities of Senna can entrain deep pancytopenia related to bone marrow toxicity as well as venous thrombosis and can be life-threatening to a child. Hence, there should be preventive sensitization of parents and medical providers. This case report illustrates the danger of prolonged and excessive use of Senna in children. Further pharmacological studies are needed to investigate age-appropriate dosages, treatment durations, and potential adverse effects to make the prescription of this medicinal plant safe.
